# Research on English Achievement Analysis Based on Improved CARMA Algorithm

**DOI:** 10.1155/2022/8687879

**Published:** 2022-01-24

**Authors:** Lin Hu

**Affiliations:** Jilin University of Finance and Economics Jilin, Changchun 130117, China

## Abstract

This paper uses data mining technology to analyze students' English scores. In view of the influence of many factors on students' English performance, the analysis is realized by using the association rule algorithm. The thesis analyzes and applies students' English scores based on association rules and mainly does the following work: (1) at present, the problem of the CARMA algorithm is low operating efficiency. The combination of the genetic algorithm's crossover, mutation, and the CARMA algorithm realizes the fast search of the algorithm. The simulation results show that the operation performance of the algorithm is greatly improved after the crossover and mutation operations in the genetic algorithm are applied to the CARMA algorithm. The simulation results show that the mining accuracy of the improved algorithm is 97.985%, and the mining accuracy before the improvement is 92.221%, indicating that the improved algorithm can improve the accuracy of mining. (2) By comparing the mining time of the improved CARMA algorithm, the traditional CARMA algorithm, the FP-Growth algorithm, and the Apriori algorithm, the results show that when the number is 6,500, the mining efficiency of the improved CARMA algorithm is twice that of the other three algorithms. As the amount of data increases, the effect of improving mining efficiency gradually increases. (3) By using the improved CARMA algorithm to analyze students' English performance, it is found that the quality of student performance is strongly related to the quality of daily homework, and if it is related to the teacher's gender, professional title, etc., it is recommended that schools should pay more attention to homework during the teaching process.

## 1. Introduction

Facing a large amount of information and data, the rapid development and wide application of data mining technology effectively solve the problem of how to use massive amounts of information. Various data mining analysis methods are also increasing. Classification, prediction, association rules, clustering, etc., are all frequently used data mining methods. Among them, association rules are widely used in big data mining as a rule-based machine learning algorithm [1]. Association rules find data with certain relevance and relevance from massive data, and describe the common attributes or laws of these data. Association rules are a simple and practical analysis technique, which originally originated from shopping basket analysis and goods placement, and increases the sales of goods by finding the connections between goods. The current association rules have been applied in many fields, such as the financial industry and e-commerce. The Apriori algorithm, CARMA algorithm, and FP-Growth algorithm are the classic algorithms in association rules [2–4].

In recent years, data mining has also been widely used in the education field, especially in the application of student performance. Through the analysis of the academic performance, it is possible to understand the student's learning situation, find the student's weaknesses, better check the deficiencies and make up for omissions, promote the improvement of students' academic performance, and at the same time improve the school's teaching level. However, the current analysis of student performance is mainly simple performance query, deletion, etc., and a series of behavioral factors related to academic performance have not been excavated and analyzed, such as the impact of students' daily behavior on learning [5]. However, it is well known that there is a close relationship between student learning behavior and academic performance, so the current role of data mining has not been fully utilized [6]. In the face of this situation, this article conducts a more in-depth analysis of the application of data mining in the analysis of high-efficiency students' English scores and specifically selects association rules to mine student scores. The level of students' English performance is also inextricably linked to factors such as the school's teachers and their teaching conditions. Therefore, it is suitable to use the association rule algorithm to find the relationship between English performance and student behavior, teachers, and other factors so as to propose English teaching suggestions.

## 2. Related Theories

### 2.1. Data Mining

**Figure 1:**
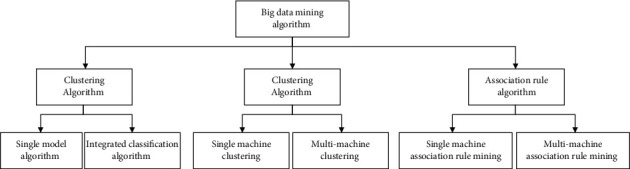
Big data mining method.

Big data [7] refers to data sets that cannot be collected, processed, and analyzed within a tolerable time frame using traditional data processing and analysis techniques or software and hardware tools. Compared with the traditional dataset, it has 4V characteristics [8]: scale (Volume), diversity (Variety), value (Value), and effectiveness (Velocity). According to different mining tasks, data mining processing methods can be divided into classification analysis, cluster analysis, association rule mining, and sequence analysis. Blackett further divides the processing methods into 3 categories according to the degree of data analysis [9]: descriptive analysis that uses historical data to summarize, predictive analysis that uses related technologies to predict future probabilities or trends, and discovers hidden data relevance to help users make a regular analysis of decision making. Cluster analysis is used to describe data, which classifies data by measuring the similarity between data; classification analysis obtains classification rules based on training data, so it is often used for predictive analysis; the purpose of association rule mining is to find and extract valuable hidden patterns, and the idea is also applicable to sequence pattern discovery. Among them, classification belongs to supervised learning, and clustering and association rule mining belong to unsupervised learning. As commonly used machine learning algorithms, these three types of methods have received more attention and research in academia, and many methods and theories have been developed. Big data mining is specific, and the algorithm definition is shown in Figure 1. This article will expand the relevant content in sequence according to classification, clustering, and association rule mining.

#### 2.1.1. Classification Algorithm

According to the mental framework in Figure 1, the classification algorithm of streaming data is first introduced. Classification is a supervised learning method [10]. Its purpose is to discover the classification rules through the examples in the training data set and the class labels to which the examples belong and use the classification rules to predict the class label of the unknown instance. The classification process has two stages: (1) the classification rule learning stage, which analyzes and summarizes the label situation of each instance class in the training set, discovers the classification rules, and creates a suitable classifier model; (2) the classification stage of the test set. In this stage, the classification accuracy of the classifier model needs to be evaluated on the validation set. If the accuracy is good, the classifier is used to preclassify the instances in the test set; otherwise, the classifier model needs to be retrained.

Streaming data classification algorithms are mainly classified into two categories according to the number of classifiers included [11] as follows: single model algorithm and integrated classification algorithm. The single-model algorithm improves and expands the traditional batch classification algorithm and is equipped with a specific concept drift processing mechanism so that it can adapt to the changes in the data in the stream. According to traditional classification algorithms, single-model algorithms can be divided into several types based on decision trees, support vector machines, and Bayesian models. The ensemble learning algorithm trains N base classifiers based on data in different time periods, combines each classification model into an ensemble classifier through a certain combination method, and synthesizes the results of each base classifier according to a certain mechanism to obtain the result of the ensemble model, making the integrated model more robust and able to deal with the problem of concept drift flexibly.

#### 2.1.2. Clustering Algorithm

Big data clustering is an unsupervised learning method [12], which does not require predefined categories and training samples. Its purpose is to divide the unknown data set into several clusters so that the data points in the same cluster are as similar as a possible degree, and the data points in different clusters have as much dissimilarity as possible. There are two main stages in the clustering process:

1. Define the similarity function and use it as the basis for judging whether the data are similar.
2. Select the appropriate clustering algorithm to divide the data objects into different clusters. Big data clustering analysis methods can be divided into single-machine clustering methods and multimachine clustering methods [13]. The realization of the single-machine clustering method is mainly through the optimization and expansion of the classic clustering algorithm and the dimensionality reduction or sampling of the data set, while the mainstream multimachine clustering method is mainly reflected in the architecture and other aspects. There is no essential difference between the class algorithm and the classic clustering algorithm.
3. Association rule algorithm big data association rule mining refers to discovering hidden information relevance from a large amount of data and analyzing the relevance to help decision makers make correct decisions. The strength of association rules can be measured by support and confidence [14]: if there is a rule X ⟶ Y, the support is the frequency of transactions *X* and Y appearing in the transaction database, which is used to judge whether the rule has utility value; confidence: this degree is the frequency of the occurrence of transaction Y when transaction *X* appears and is used to judge the correctness of the rule. There are two main steps in association rule mining: (1) find out the rule whose support degree is not less than the minimum support degree from the data called “frequent pattern” or “frequent item set;” (2) find out the confidence degree not less than the minimum from the frequent pattern The strong association rules with confidence thresholds focus on the mining of frequent patterns. Association rule mining can be divided into basic single-machine association rule mining and multimachine parallel association rule mining based on the distributed computing framework [15].

### 2.2. Association Rule Method

Association rules refer to the frequent patterns, correlations, or causal structures that exist between item sets or object sets in log data, relational data, or other information carriers. The acquisition of association rules is mainly through data mining methods to find frequent patterns from a large number of event record databases [16] and discover new patterns and potential knowledge by analyzing frequent situations.

#### 2.2.1. Algorithm Type

(1) Apriori algorithm. The Apriori algorithm based on frequent item sets is one of the classic algorithms in the field of association rule mining [17]. The data structure of the Apriori algorithm is simple, clear, and easy to understand, but the application of the Apriori algorithm requires multiple scans of the database, which is expensive. Although the Apriori algorithm itself has made some optimizations, the time and space complexity of using this method is large, and the efficiency is not high. The specific steps are as follows:

1. Scan the original transactional data and extract the candidate 1 item set. According to the given minimum support *s*, extract the item set whose frequency of the item set is not less than the support *s* to form a frequent 1 item set.
2. Scan the original transactional data and extract the set of candidate 2 item sets, judge each 2 item sets in the set, exclude the 2 item sets that do not contain frequent 1 item set, and form a new set as the candidate set. According to the support degree *s*, the frequent 2 item sets are filtered out.
3. Repeat the idea of the second step, get frequent 3 item sets, frequent 4 item sets,...frequent *n* item sets, and *n*+1 item sets do not meet the support threshold condition.
4. Given the confidence level, judge whether each frequent item set rule is a valid rule, calculate the lift of the rule, and judge whether the strong rule is valid.

**Figure 2:**
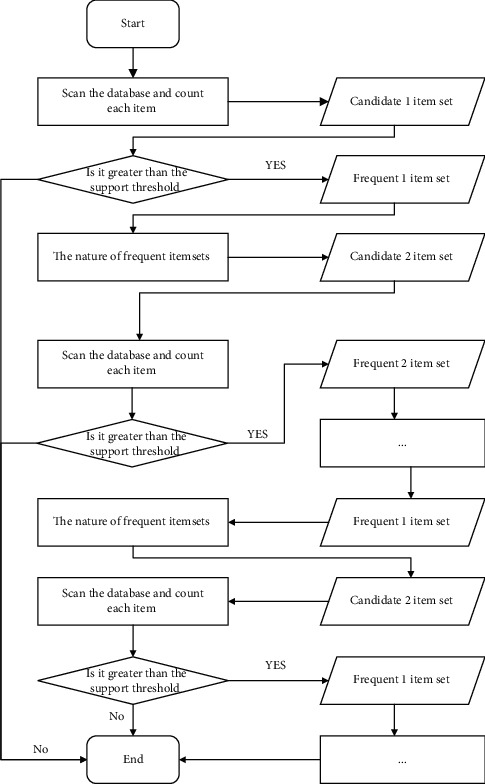
Schematic diagram of Apriori algorithm.

The steps of the Apriori algorithm are shown in Figure 2.

(2) FP-Growth algorithm. The advantage of the FP-Growth algorithm is that it mines all frequent item sets without generating a large number of candidate sets. With the generation of large-scale data, the processing capabilities of data mining in a single-machine environment are challenged in both storage and computing. A parallel computing environment has become the first choice for solving big data processing problems. Some researchers have proposed parallel algorithms based on multithreading [18]. Although the pressure of storage and calculation has been relieved to a great extent, the limitation of memory resources has become the algorithm of the bottleneck of expansion; the FP-Growth algorithm is based on the Apriori principle. It finds frequent item sets by storing the data set on the FP (Frequent Pattern) tree but cannot find the association rules between the data. The FP-Growth algorithm only needs to scan the database twice, while the Apriori algorithm needs to scan the data set once when seeking each potential frequent item set. The process of the FP-Growth algorithm is to first construct the FP tree and then to use it to mining frequent item sets. When constructing the FP tree, you need to scan both sides of the data set. The first scan is used to count the frequency, and the second scan is to consider frequent item sets.

**Figure 3:**
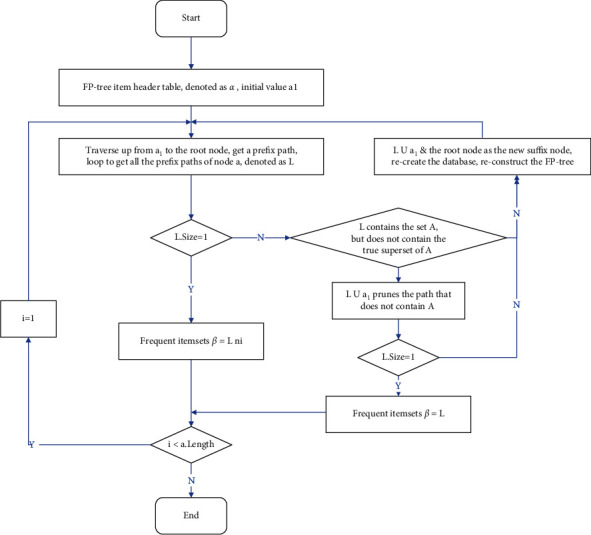
Flowchart of FP-Growth algorithm.

The steps of the FP-Growth algorithm are shown in Figure 3.

(3) CARMA algorithm. The CARMA algorithm was proposed by Christian Hidber and is a classic algorithm in association rules. It uses the iterative method of layer-by-layer search to find the relationship between the item sets in the database to form rules. The process consists of connection (matrix-like operations) and pruning (removing unnecessary intermediate results). The concept of item sets in this algorithm is a collection of items. A set containing K items is a K-item set. The frequency of item set occurrence is the number of transactions containing the set, which is called the frequency of the itemset. If a certain item set meets the minimum support degree, it is called a frequent item set [19].

The CARMA algorithm is mainly to find the set sum of the data items in the transaction set *D* frequently. It has mainly two steps. In the first step of the application process, a superset of the data item frequency set will appear. This set is a set of potential data item frequency sets. The second step may not be performed on all transaction sets. Traverse, but since the user number can modify the support in the first step, the result of the algorithm after the second step should match the support modified in the first step. In the entire application process, the algorithm realizes the maintenance of the set V, which can process each transaction in D and add or delete elements in the set V. When the second traversal is completed, V is the final result.

#### 2.2.2. Algorithm Evaluation Criteria

Data association processing can also be called “data analysis” or “data association analysis.” Association analysis is a simple practical data analysis technology is used to discover correlations or associations in a large number of data sets so as to describe the laws and patterns of certain attributes in a thing at the same time. Association rule analysis is generally used. Association rule discovery is to find the association between things. For example, it can be found from a set of data items included in a set of (assuming commodity purchases) transactions as follows: if a purchase transaction contains item *X* and item Y, then N% of all purchase transactions includes item *Z* of the purchase transaction. Sequence rule discovery is similar to association rule discovery, which is used to find the sequence relationship between things.

The proportion of transactions that include both pre-events and postevents in the association rules is called confidence; the proportion of transactions that include both pre- and postrules is called support.

The calculation method of confidence is

(1) $$\begin{array}{c} \text{Confidence}\left(a\Rightarrow b\right)=p\left(\frac{b}{a}\right). \end{array}$$

The calculation method of support is

(2) $$\begin{array}{c} \text{Support}\left(a\Rightarrow b\right)=p\left(a\cup b\right). \end{array}$$

## 3. Improving the CARMA Algorithm

The CARMA algorithm is a classic association rule algorithm, which has low memory usage and continuous data processing features. However, the CARMA algorithm is not fast, and the mining efficiency is low. The way of solving this problem is one difficult question. This section proposes to introduce the genetic algorithm into the CARMA algorithm to improve the accuracy of the algorithm.

### 3.1. Basic Principles

**Figure 4:**
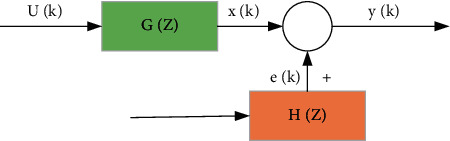
Structure diagram of random model.

The CARMA algorithm is derived from the system model. A system model refers to a model that includes inputs and outputs, including a random model and a deterministic model. Random models can be seen everywhere in practice; this is due to the randomness of the system due to noise interference [20].  Figure 4 shows the structure of the random model.

The stochastic system can be expressed by the following formula:

(3) $$\begin{array}{c} A\left(z\right)y\left(k\right)=B\left(z\right)u\left(k\right)+\frac{D\left(z\right)}{C\left(z\right)}v\left(k\right),\\ y\left(k\right)=\frac{B\left(z\right)u\left(k\right)}{A\left(z\right)}+\frac{D\left(z\right)}{A\left(z\right)C\left(z\right)}v\left(k\right),\\ \text{Set }G\left(z\right)=\frac{B\left(z\right)}{A\left(z\right)},\;\\ H\left(z\right)=\frac{D\left(z\right)}{A\left(z\right)C\left(z\right)}. \end{array}$$

In the definition symbol, *G*(*z*) is called the system model transfer function, and *H*(*z*) is called the noise model transfer function.

When *C*(*z*)=*D*(*z*),  *u*(*k*)=0, we get *A*(*z*)*y*(*k*)=*B*(*z*)*u*(*k*)+*v*(*k*); this is called the “autoregressive model.”

When *C*(*z*)=1 and *u*(*k*)=0, *A*(*z*)*y*(*k*)=*B*(*z*)*u*(*k*)+*D*(*z*)*v*(*k*) is obtained, which is called the “controlled CARMA model.”

The CARMA algorithm is an online algorithm, which consumes less memory during the calculation process, so it is widely used in online mining. However, the disadvantage of this algorithm is that it runs slightly slower. In order to improve the efficiency of the algorithm, this paper proposes to combine the genetic algorithm and the CARMA algorithm to form the CARMA algorithm based on the genetic algorithm to improve the efficiency of the algorithm.

### 3.2. Genetic Algorithm

**Figure 5:**
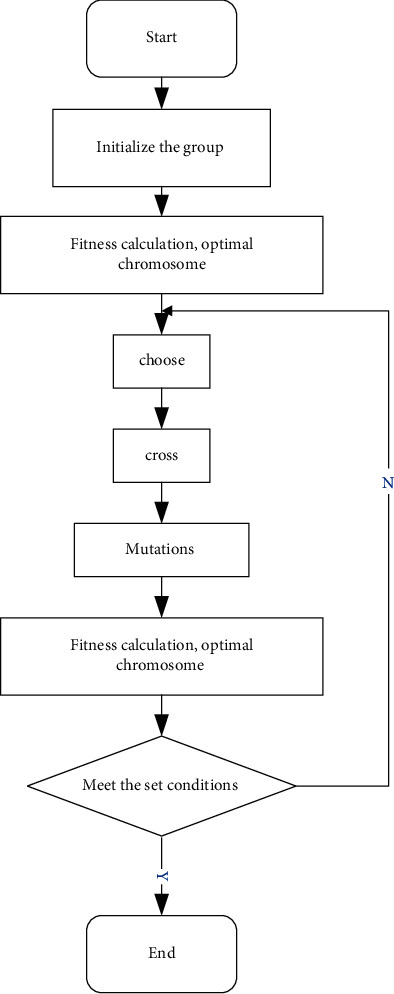
Flowchart of genetic algorithm.

Genetic algorithm is a bionic algorithm born out of natural selection in Darwin's theory of evolution [21]. The algorithm flow of the process diagram is shown in Figure 5.

It can be seen from the figure that the gene is the most basic unit in the structure of the genetic algorithm, and the gene constitutes the chromosome and finally constitutes the population. A detailed analysis is given below:

(1) Gene: In biology, a gene is a nucleotide sequence that produces a polypeptide chain. The DNA that contains genetic information in this sequence is the smallest unit of genetic behavior. In order to simulate gene behavior, the concept of a gene is added to the genetic algorithm [22].
(2) Chromosomes: According to the principles of genetics, chromosomes are composed of a limited number of genes, and chromosomes carry a lot of information. There are two ways to encode chromosomes in genetic algorithms:
One is binary numbering, and the other is cross coding. The following two pairs of codes are analyzed. The principle of binary encoding is to convert design variables into binary encoding. Assuming that there are variables (1, 2, ...) *p*_*i*_ *i*=*n*, then the corresponding binary string length is set to (1, 2, ...) *l*_*i*_ *i*=*n*, and the decimal value (1,2, ...) *M*_*i*_ *i*=*n*. The calculation method is
  (4) $$\begin{array}{c} M_{i}=\frac{\left(2^{l_{i}}-1\right)\left(p_{i}-a_{i}\right)}{b_{i}-a_{i}}. \end{array}$$
(3) Population: In order to simulate the biological population, a limited number of chromosomes are integrated as the population number in the genetic algorithm. The population size setting directly affects the efficiency of the algorithm. The population size of the genetic algorithm designed in this paper is set in 150 pieces.
(4) Fitness: a fitness function is introduced for the evaluation of indicators. The fitness calculation methods are as follows: when calculating the maximum value of the objective function, the method is calculated according to the following formula:
  (5) $$\begin{array}{c} \text{Fit}\left(f\left(x\right)\right)=f\left(x\right). \end{array}$$
The calculation method of the minimum value of the objective function is calculated according to the following formula:
  (6) $$\begin{array}{c} \text{Fit}\left(f\left(x\right)\right)=-f\left(x\right). \end{array}$$
Variant calculation method for solving the objective function optimization problem is as follows: when the minimum value needs to be calculated, there are
  (7) $$\begin{array}{c} \text{Fit}\left(f\left(x\right)\right)=\left\{\begin{array}{c} c_{\max}-f\left(x\right)\;f\left(x\right)<c_{\max}\\ 0f\left(x\right)\geq c_{\max} \end{array}\right., \end{array}$$
where *c*_max_ represents the maximum estimated value of the function. When the objective function is the maximum, then
  (8) $$\begin{array}{c} \text{Fit}\left(f\left(x\right)\right)=\left\{\begin{array}{c} -c_{\min}+f\left(x\right)\;f\left(x\right)>c_{\min}\\ 0\;f\left(x\right)\leq c_{\min} \end{array}\right., \end{array}$$
where *c*_min_ represents the minimum estimated value of the function. The objective function fraction calculation algorithm is as follows: when the minimum problem needs to be calculated, there are
  (9) $$\begin{array}{c} \text{Fit}\left(f\left(x\right)\right)=\frac{1}{1+f\left(x\right)+c}\;c\geq 0,\;c+f\left(x\right)\geq 0. \end{array}$$
When the objective function calculates the biggest problem, there are
  (10) $$\begin{array}{c} \text{Fit}\left(f\left(x\right)\right)=\frac{1}{1-f\left(x\right)+c}\;c\geq 0,\;c-f\left(x\right)\geq 0. \end{array}$$
The fitness function changes mainly include linear changes, power function changes, and exponential changes.
(5) Selection: The genetic algorithm introduces selection to select the superior individual from the group, and the elimination of inferior individuals makes the population better. At present, the selection of calculation methods in genetic algorithms is mainly based on deviation, expectation, probability, and so on. This article chooses according to the compass calculation method. The probability calculation method is as follows:
  (11) $$\begin{array}{c} P_{t}=\frac{f_{t}}{\sum_{i=1}^{M}f_{t}}. \end{array}$$
(6) Crossover: It refers to the gene exchange between two parents, and the algorithm search ability is enhanced after crossover.
(7) Mutation: It is easy for genes to crossover all combinations during the crossover process. In order to prevent the partial algorithm, the optimal case introduces variation. The mutation rate in the genetic algorithm cannot be set too high. If it is too high, the algorithm will no longer have the search ability.

### 3.3. Improved CARMA Algorithm Model

**Figure 6:**
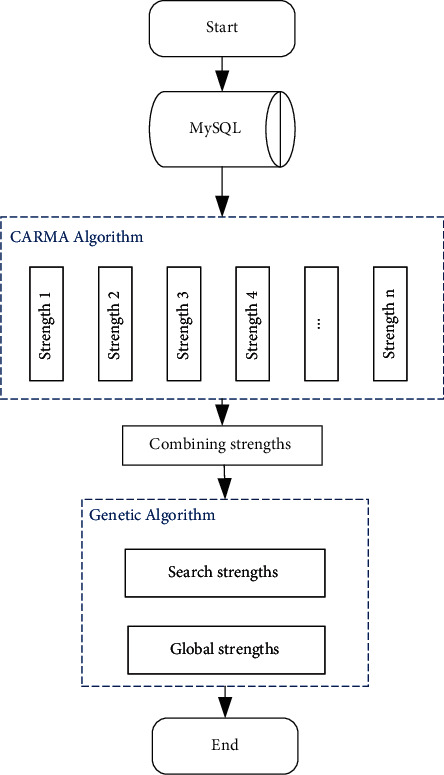
CARMA algorithm flowchart based on genetic algorithm.

The principle of the CARMA algorithm based on the genetic algorithm proposed in this paper is to use the genetic algorithm for frequent item search. However, the CARMA algorithm only implements mining, which reduces the operating efficiency of the CARMA algorithm. The way to improve the efficiency of the CARMA algorithm is to constrain the crossover operator and mutation algorithm of the genetic algorithm, which improves the algorithm operation efficiency. According to the CARMA algorithm idea for algorithm design [23], the designed algorithm based on a genetic algorithm, i.e., the CARMA algorithm flow, is shown in Figure 6.

From Figure 6, we can see that the improved CARMA algorithm first reads student information from the MySQL database and then divides the data in the MySQL database into several sub-data sets and stores them in the memory; second, it uses the rules in the CARMA algorithm to control the data in the memory database. Strong decomposition is carried out; the decomposed data are merged; the genetic algorithm is applied to the CARMA algorithm to achieve strength search.

From Figure 6, we can see that the core idea of the algorithm consists of two parts: (1) the MySQL database is split into multiple subdatabases, the data are transferred to the memory, and then, the operation of merging the strength set is implemented; (2) the genetic algorithm is introduced into the strength set to find the optimal strength set. The use of the genetic algorithm greatly reduces data I/O operations, thereby improving the operating efficiency of the CARMA algorithm. The fitness function setting is performed after completing the encoding according to the above operations. When genetic algorithms need to solve problems, the CARMA algorithm reads the database efficiency problem. For this reason, the fitness function constructed in this paper includes confidence and support as two variables of persistence. The calculation method of the fitness function is as follows:

(12) $$\begin{array}{c} f\left(x\right)=aS\left(x\right)+bB\left(x\right). \end{array}$$

In the above formula, *a* and *b* are constants, *S*(*x*) represents the degree of support, and *B*(*x*) represents the degree of confidence. Next, the genetic algorithm selection operation is performed. As analyzed above, this article chooses the probability calculation method to choose. Algorithm 1 describes the steps of the CARMA algorithm based on the genetic algorithm in detail.

## 4. Experiment

### 4.1. Experimental Data and Operating Environment

The experimental system configuration environment is the operating system Windows7 64-bit Professional Edition; memory is 16 GB (16 GB × 1) DDR42666 MHz; hard disk is SSD256 G; CPU frequency is 2.2 GHz, six cores/twelve threads, simulation level; the platform is MATLAB7.0.

Data source: the experimental data come from the database stored in the student status management system of a school in the past three years (the student status management system database is an open-source MySQL database). At present, the amount of data stored in the student status management system reaches 500 Mb. The first step is to divide the database. This algorithm is divided into 20 data sets. The data are read into the memory, respectively, and the strength set is calculated according to the CARMA algorithm, and the strength set is combined together.

There are many attributes in the original four-level performance data, and attributes that are irrelevant, weakly relevant, or redundant with the research are deleted. In the end, the attributes we selected are the four-level total score (S), the total test score (T), the listening score (L), the reading score (R), and the writing score (W). According to the previous analysis of the student's related information factors, the related table is obtained. Then, the parameters are set; the fitness function method used is *f*(*x*) = *S*(*x*) + *B*(*x*); the minimum support is 0.21, the minimum confidence is 0.81; the initial number of the population is set to 150; the mutation rate is 0.14; the crossover probability is 0.9.

### 4.2. Experimental Results

From Table 1, it can be seen that according to the combination of the genetic algorithm and the CARMA algorithm, the student performance mining found that there is a correlation between student absenteeism, usual performance, family status, and English performance.

**Figure 7:**
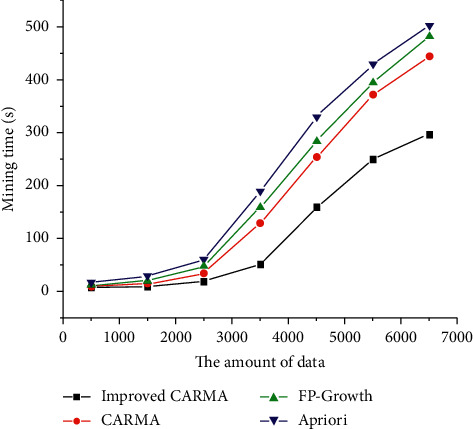
Impact of data volume on mining efficiency.

The performance analysis of the improved algorithm and the CARMA algorithm is as follows. The number of excavations affects the efficiency of excavation. The memory is 512 Mb, the number of division levels is 6, and the algorithm is realized by MATLAB programming. The result is shown in Figure 7.

It can be seen from Figure 7 that the improved algorithm and the classic CARMA algorithm increase the mining time as the number of mining increases. The operating efficiency of the improved CRRMA algorithm has been improved, which is better than the improved algorithm in literature [24] and better than the classic CARMA algorithm, which shows that adding the genetic algorithm to an algorithm can improve the operating efficiency of the algorithm.

In order to investigate the accuracy of the improved algorithm in data mining, the existing data are obtained from the student information database. Mining data quality analysis is performed according to the formula as follows:

(13) $$\begin{array}{c} \eta =\frac{a}{a+b}\%. \end{array}$$

In the above formula, *η* represents the mining success rate, a represents the number of successful mining, and *b* represents the number of failed mining. The excavation results are shown in Table 2.

It can be seen from Table 2 that the mining accuracy rate of the improved algorithm reaches 97.985%, and the mining accuracy rate before the improvement is 92.221%, indicating that the improved algorithm can improve the accuracy of mining.

## 5. Conclusion

This paper uses data mining technology to analyze students' English scores. Aiming at the influence of many factors on the students' English scores, the analysis is realized by using the association rule algorithm. This paper is based on association rules. The students' English scores are analyzed and applied, and the main tasks are as follows:

1. At present, the problem of the CARMA algorithm is low operating efficiency. The combination of the genetic algorithm's crossover, mutation, and CARMA algorithm realizes the fast search of the algorithm. The simulation results show that the operation performance of the algorithm is greatly improved after the crossover and mutation operations in the genetic algorithm are applied to the CARMA algorithm. The simulation results show that the mining accuracy of the improved algorithm is 97.985%, and the mining accuracy before the improvement is 92.221%, indicating that the improved algorithm can improve the accuracy of mining.
2. By comparing the mining time of the improved CARMA algorithm, the traditional CARMA algorithm, the FP-Growth algorithm, and the Apriori algorithm, the results show that when the number is 6,500, the mining efficiency of the improved CARMA algorithm is twice that of the other three algorithms. As the amount of data increases, the effect of improving mining efficiency gradually increases.
3. By using the improved CARMA algorithm to analyze students' English performance, it is found that the quality of student performance is strongly related to the quality of daily homework, and if it is related to the teacher's gender, professional title, etc., it is recommended that schools should pay more attention to homework during the teaching process.

## Figures and Tables

**Algorithm 1:**
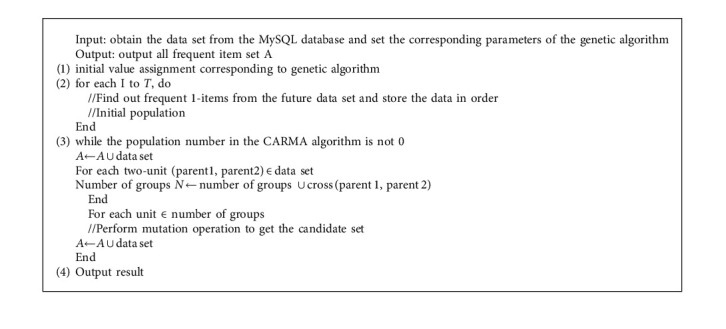
CARMA algorithm steps based on genetic algorithm.

**Table 1:** Part of student's English performance-related collection.

Number	Rule	Support	Confidence
1	The title of teacher and the quality of class (good grades)	0.342	0.859
2	The gender of teacher and the number of homework (average grades)	0.453	0.871
3	Teacher's length of service and degree of the course (good grades)	0.234	0.882
4	The quality of class and other subjects (average grades)	0.512	0.832
5	Student absent and Daily test (bad grades)	0.389	0.912
6	The number of homework and Family status (excellent grades)	0.276	0.903

**Table 2:** Comparison of improved algorithm and CARMA algorithm mining quality.

	CARMA algorithm	Improved CARMA algorithm
Number of successes	10,006	10,023
Number of failures	844	202
Success rate	92.221	97.985
Failure rate	7.779	2.015

## Data Availability

The dataset can be accessed upon request.
